# Do Nutritional Status and Dietary Intake Influence the Ghrelin Response?

**DOI:** 10.3390/nu18193271

**Published:** 2026-10-04

**Authors:** Marcelina Radziszewska, Joanna Smarkusz-Zarzecka, Urszula Cwalina, Beata Zyśk, Angelika Buczyńska-Backiel, Monika Moroz, Joanna Roszkowska, Monika Stołyhwo-Gofron, Edyta Adamska-Patruno, Adam Krętowski, Lucyna Ostrowska

**Affiliations:** 1 Department of Dietetics and Clinical Nutrition, Medical University of Bialystok, 15-054 Bialystok, Poland; joanna.smarkusz-zarzecka@umb.edu.pl (J.S.-Z.); beata.zysk@umb.edu.pl (B.Z.); lucyna.ostrowska@umb.edu.pl (L.O.); 2 Department of Biostatistics and Medical Informatics, Medical University of Bialystok, 15-295 Bialystok, Poland; urszula.cwalina@umb.edu.pl; 3 Clinical Research Centre, Medical University of Bialystok, 15-276 Bialystok, Poland; angelika.buczynska@umb.edu.pl (A.B.-B.); adam.kretowski@umb.edu.pl (A.K.); 4 Department of Endocrinology, Diabetology and Internal Medicine, Medical University of Bialystok, 15-276 Bialystok, Poland; 5 Clinical Research Support Centre, Medical University of Bialystok, 15-276 Bialystok, Poland; monika.moroz@umb.edu.pl (M.M.); joanna.roszkowska@umb.edu.pl (J.R.); edyta.adamska-patruno@umb.edu.pl (E.A.-P.); 6 Mediart Obesity and Overweight Treatment Clinic, 80-298 Gdansk, Poland; stolyhwo.gofron@gmail.com; 7 Department of Nutriomics, Clinical Research Centre, 15-276 Bialystok, Poland

**Keywords:** ghrelin, appetite regulation, energy homeostasis, DXA, energy intake, macronutrient intake, postprandial response, adipose tissue distribution, obesity phenotype

## Abstract

Background/Objectives: Ghrelin is a key orexigenic hormone involved in the regulation of appetite and energy homeostasis, and its fasting circulating concentrations may reflect long-term energy balance and nutritional status. The aim of the study was to assess the associations between habitual daily energy and macronutrient intake from a conventional diet and the fasting total ghrelin concentrations in individuals with different nutritional status and to evaluate the postprandial ghrelin response following consumption of a standardized meal. An additional aim was to identify body composition parameters predictive of ghrelin concentrations. Methods: The prospective study included 76 individuals aged 40–60 years, classified into four body mass index (BMI) groups. Body composition was assessed using dual-energy X-ray absorptiometry (DXA). Energy and macronutrient intake was evaluated based on standardized 3-day food records. Ghrelin concentrations were measured in the fasting state and at 60, 120, 180, and 240 min after consumption of a standardized mix liquid meal (Nutridrink Protein, 459 kcal). Results: Fasting total ghrelin concentrations were not associated with recent energy or macronutrient intake. In women, changes from the baseline differed across BMI groups at 60 and 120 min, whereas no between-group differences were observed in men. In the regression analyses, BMI and gynoid region fat showed significant coefficients in women, although the overall model was not significant, while gynoid region fat was significantly associated with fasting ghrelin in men. Conclusions: Fasting total ghrelin appears to reflect longer-term metabolic phenotype and adipose tissue distribution rather than short-term variation in habitual energy or macronutrient intake. The BMI-dependent differences in the early postprandial response observed in women further suggest that nutritional status may modify dynamic ghrelin regulation in a sex-specific manner. These findings indicate that ghrelin should not be interpreted solely as an acute marker of dietary intake, but rather within the broader context of body composition, fat distribution, and sex-related metabolic regulation.

## 1. Introduction

Food intake, the maintenance of energy homeostasis, and the regulation of metabolic processes result from the interplay between peripheral and central signals [1,2]. Eating behavior is influenced by psychological, social, and cultural factors; however, the neurohormonal system plays a central role in the regulation of hunger, satiety, and energy homeostasis [3,4]. The hypothalamus is a central component of this regulatory system, integrating signals from the gastrointestinal tract, adipose tissue, pancreas, and the central nervous system [1,2].

The gut–brain axis constitutes a bidirectional communication system between the gastrointestinal tract and the central nervous system [4,5]. Mechanical and chemical signals related to the composition and energy content of food are transmitted to the brain, including via vagal afferent fibers [4,6,7]. Appetite regulation involves both homeostatic mechanisms and motivational and hedonic processes associated with the reward system [8,9].

Disruption of the balance between homeostatic and hedonic signals may lead to altered patterns of food intake [7,9]. Gastrointestinal hormones play a key role in conveying information about energy status to the central nervous system, with ghrelin acting as a major orexigenic signal promoting food-seeking behavior and food intake [10,11,12].

Ghrelin is a 28-amino-acid peptide hormone synthesized primarily in the stomach and released under conditions of energy deficit [13,14]. It stimulates appetite and promotes meal initiation and is therefore commonly referred to as the “hunger hormone” [4,12,15]. It also participates in the regulation of the hypothalamic–pituitary axis and glucose and lipid metabolism, underscoring its broader metabolic significance [16]. Its involvement in the pathogenesis of metabolic and endocrine disorders has also been suggested [17,18,19].

Two forms of ghrelin circulate in the bloodstream: acylated and desacyl ghrelin [20]. In clinical studies, total ghrelin concentrations are most commonly measured as an indicator of overall ghrelin system activity [16]. Ghrelin concentrations increase during fasting and before meals and are suppressed after food intake, reflecting mechanisms involved in appetite regulation [4,15]. Disruption of this pattern may indicate impaired appetite control [12].

Ghrelin concentrations may vary according to nutritional status and body composition. Individuals with normal body weight tend to exhibit higher fasting ghrelin concentrations and a more pronounced postprandial decline [16,21]. Individuals with obesity generally exhibit lower fasting ghrelin concentrations and an attenuated postprandial suppression, which may represent an adaptive response to chronic positive energy balance [22,23]. Ghrelin concentrations tend to decrease further with increasing severity of obesity [21,23].

Weight loss is associated with an increase in ghrelin concentrations, which may represent an adaptive mechanism aimed at restoring energy reserves and is consistent with the set-point model [24,25,26]. The increase in ghrelin concentrations following weight loss may hinder long-term weight maintenance [27,28]. In contrast, the settling-point model proposes that body weight stabilizes as a result of environmental and behavioral influences [29,30]. In undernutrition, elevated ghrelin concentrations are observed, reflecting enhanced orexigenic signaling [31].

Both fasting ghrelin concentrations and their postprandial dynamics are important in appetite regulation. Assessing changes in ghrelin concentrations following a controlled energy load provides insight into ghrelin system function and its associations with body composition and dietary intake [4,32]. The aim of the study was to assess the association between habitual energy and nutrient intake from a conventional diet and the fasting total ghrelin concentrations in individuals with different nutritional status, as well as to analyze postprandial changes in ghrelin concentrations following the consumption of a standardized meal. An additional aim was to evaluate the associations between body composition parameters and baseline ghrelin concentrations.

## 2. Materials and Methods

### 2.1. Study Design and Study Population

The study was designed as a prospective observational-interventional study. It was conducted in accordance with a protocol approved by the Bioethics Committee of the Medical University of Bialystok (No. APK.002.94.2024). All participants received comprehensive information about the study procedures and their right to withdraw at any stage and subsequently provided written informed consent to participate. The study was registered at ClinicalTrials.gov (NCT07627802). A total of 80 participants were enrolled. Four participants discontinued the study procedure following consumption of the standardized test meal; therefore, the final analytical sample consisted of 76 participants. The final study population was classified into four BMI groups, with 19 participants in each category.

### 2.2. Inclusion and Exclusion Criteria

Women and men aged 40–60 years with a BMI ≥18.5 kg/m^2^ were eligible for inclusion. Participants had no contraindications to DXA assessment, blood sampling, or consumption of the test meal. Individuals with diagnosed disorders of glucose metabolism, endocrine diseases, renal or hepatic failure, or clinically significant gastrointestinal diseases were excluded from the study. Exclusion criteria also included a history of gastrointestinal or bariatric surgery and the use of medications or supplements that could affect metabolic or hormonal homeostasis. Additional exclusion criteria were pregnancy, lactation, and other diseases or clinical conditions that could significantly interfere with the interpretation of the study results.

### 2.3. Assessment of Anthropometric Parameters

Anthropometric measurements, including body weight and height, were obtained for all participants. Measurements were performed at the Population Research Center of the Medical University of Bialystok using an InBody 770 body composition analyzer (InBody Co., Ltd., Seoul, South Korea) and a SECA 217 stadiometer (seca GmbH & Co. KG, Hamburg, Germany). Measurement accuracy was 0.1 kg for body weight and 0.5 cm for height. Based on these measurements, body mass index (BMI) was calculated and used to classify participants into four groups:

- G0—normal body weight (18.5–24.9 kg/m^2^; *n* = 19),
- G1—overweight (25.0–29.9 kg/m^2^; *n* = 19),
- G2—class I obesity (30.0–34.9 kg/m^2^; *n* = 19),
- G3—class II and III obesity (≥35.0 kg/m^2^; *n* = 19).

### 2.4. Body Composition Analysis Using DXA

Body composition was assessed in all participants using dual-energy X-ray absorptiometry (DXA). The examination was performed in the fasting state, with participants in the supine position, at the Population Research Center of the Medical University of Bialystok using a GE Lunar iDXA densitometer (GE Healthcare, Madison, WI, USA). Participants were instructed to avoid strenuous physical activity for 24 h before the examination and on the day of the assessment, and to remove all metal objects before the scan. DXA enables the assessment of total fat mass, lean body mass, bone mineral parameters, and regional fat distribution, including visceral adipose tissue (VAT) and subcutaneous adipose tissue (SAT) area. Resting metabolic rate (RMR) was estimated using the Mifflin–St Jeor equation, as implemented in the enCORE DXA software (GE Healthcare, Madison, WI, USA). To characterize the study population and compare anthropometric and body composition parameters between women and men, the following variables were analyzed: height, body weight, BMI, the proportion of fat mass in the android and gynoid regions, VAT and SAT area, and the android-to-gynoid (A/G) ratio. Android and gynoid adiposity were expressed as regional percent fat (% fat) as provided by the DXA software. The android-to-gynoid (A/G) ratio was calculated as the ratio of android to gynoid percent fat. Participant characteristics are presented in Table 1.

### 2.5. Food Records

Participants completed a 3-day food record covering two weekdays and one non-working day during the week preceding the study visit. Before completing the records, participants were instructed on how to document their dietary intake. They recorded all food and beverages consumed, together with meal times, portion sizes, and methods of food preparation. Portion sizes were estimated using household measures; some participants were also able to provide the weight of selected food items. During the study visit, members of the research team reviewed the records for completeness and accuracy and clarified any uncertainties. The food records were subjected to quantitative analysis. For each participant, the 3-day mean daily energy intake (kcal) and mean daily intake of protein, fat, and carbohydrates (g/day) were calculated. Nutrient intake was analyzed using Dieta 6 software (National Institute of Public Health NIH—National Research Institute, Warsaw, Poland). Analysis of the food records enabled an assessment of the potential association between current energy and macronutrient intake from the habitual diet and fasting total ghrelin concentrations, according to sex and nutritional status.

### 2.6. Blood Sampling Procedure

Venous blood samples were collected from all participants for the measurement of total ghrelin concentrations. Blood sampling was performed in the morning after an overnight fast of at least 12 h at the Clinical Research Support Center of the Medical University of Bialystok. The procedure was conducted on the same day as the DXA examination to ensure standardized study conditions. Blood was collected from the antecubital vein using standard aseptic procedures. Approximately 10 mL of blood was obtained, of which 2.7 mL was allocated for the measurement of total ghrelin concentrations. The remaining biological material was appropriately processed and stored in accordance with the study protocol for subsequent laboratory analyses. The first blood sample was collected in the fasting state to determine baseline ghrelin concentrations. Participants then consumed 187.5 mL of a standardized liquid test meal (Nutridrink Protein; Nutricia, Warsaw, Poland), the composition of which is presented in Table 2.

Subsequent blood samples were collected at 60, 120, 180, and 240 min after consumption of the test meal, allowing for an assessment of the postprandial changes in total ghrelin concentrations. All blood samples were collected under controlled conditions by the same medical personnel to ensure procedural consistency and reproducibility.

Four participants experienced a deterioration in well-being after consumption of the standardized test meal, which precluded safe continuation of the study procedures. These participants were withdrawn from further participation in the protocol and received appropriate medical care. The final analysis included 76 participants.

### 2.7. Laboratory Analysis of Total Ghrelin Concentrations

Biochemical analyses were performed at the Laboratory of the Department of Endocrinology, Diabetology, and Internal Medicine Medical University of Bialystok. Biological samples were collected and prepared for analysis in accordance with the manufacturer’s instructions for the laboratory assay kits. After all samples had been collected as part of the experimental procedure, they were appropriately processed and stored at approximately −80 °C until laboratory analysis. For the measurement of total ghrelin concentrations, 2.7 mL of venous blood was collected into pre-chilled (4–8 °C) K2EDTA blood collection tubes containing a proprietary cocktail of protease inhibitors, specifically optimized for the stabilization of metabolic markers under the name BD P800 Blood Collection System (BD Biosciences, Franklin Lakes, NJ, USA). The samples were immediately centrifuged at 1300 rpm for 10 min at 4 °C. Plasma was then separated from the cellular fraction, aliquoted into Eppendorf tubes, and stored at −80 °C until analysis. Total ghrelin concentrations were measured using a competitive radioimmunoassay (RIA) with commercially available Ghrelin (Total) RIA kits (GHRT-89HK, Millipore, St. Charles, MI, USA). The assay is based on competition between the ^125I-labeled antigen and ghrelin present in the sample for binding to a specific antibody. Following precipitation of the antigen–antibody complex and the removal of unbound antigen, radioactivity was measured using a Wallac Wizard 1470 Automatic Gamma Counter (PerkinElmer, Turku, Finland). Ghrelin concentrations were determined from a standard curve generated using the standards provided by the manufacturer.

### 2.8. Statistical Analysis

Statistical analyses were performed using Statistica (version 13.3; TIBCO Software Inc., Palo Alto, CA, USA) and R (version 4.3.1; R Foundation for Statistical Computing, Vienna, Austria). Normality of data distribution was assessed using the Shapiro–Wilk test and visual inspection of histograms and Q–Q plots. Given the distributional characteristics of the variables and the small sample sizes within BMI-stratified subgroups, non-parametric methods were applied for descriptive between-group comparisons, whereas regression and mixed-effects models were used for the primary analyses. Data are presented as medians with interquartile ranges (Q1–Q3) or model-based estimates with 95% confidence intervals, as appropriate.

Differences between women and men in baseline anthropometric and body composition parameters were evaluated using the Mann–Whitney U test. Fasting total ghrelin concentrations were initially compared across BMI groups using the Kruskal–Wallis test, with post hoc pairwise comparisons performed using Dunn’s test with Bonferroni correction when the overall test indicated statistical significance.

To address the primary objective—assessing the association between habitual energy and nutrient intake and fasting total ghrelin concentrations in relation to nutritional status—multiple linear regression models were fitted with fasting total ghrelin as the dependent variable and BMI category and mean daily carbohydrate intake (centered) as predictors. An additive model including BMI status and carbohydrate intake was first estimated, followed by a model including the carbohydrate intake × BMI-status interaction to test whether the association between carbohydrate intake and ghrelin differed across BMI categories. Model comparison was performed using likelihood ratio tests (analysis of variance for nested models), and simple-slope analyses were conducted to estimate BMI-specific carbohydrate–ghrelin slopes. Pairwise comparisons of slopes between BMI groups were adjusted for multiple testing using the Holm procedure. These regression analyses were specified a priori as parsimonious, biologically motivated models; more complex sex-stratified models and extended predictor sets were considered exploratory and are reported in the Supplementary Materials.

To address the secondary objective—analyzing postprandial changes in ghrelin concentrations following consumption of the standardized meal in relation to nutritional status—linear mixed-effects models were used. Ghrelin concentrations were log-transformed to improve model fit and residual diagnostics. Models included fixed effects for time (0, 60, 120, 180, and 240 min) and BMI category, with a random intercept for participant (patient_id) to account for within-subject correlation of repeated measures. An additive model with main effects of time and BMI status was first estimated, followed by a model including the time × BMI-status interaction. Model comparison was performed using likelihood ratio tests. Estimated marginal means and contrasts for changes from the baseline (time vs. 0 min) within each BMI group were obtained using the emmeans package, with Holm adjustment for multiple post hoc comparisons. Results from the interaction model are interpreted as exploratory, given the non-significant omnibus comparison between additive and interaction models.

Given the relatively small subgroup sizes and the large number of potential comparisons, the risk of type I error was explicitly addressed by: (i) limiting the number of primary models to parsimonious, hypothesis-driven analyses focused on nutritional status; (ii) applying Bonferroni or Holm corrections for multiple post hoc tests; and (iii) distinguishing confirmatory analyses (primary regression and mixed-effects models) from exploratory analyses (sex-stratified models and extended predictor sets). A two-sided *p*-value < 0.05 was considered statistically significant for primary analyses; exploratory findings are interpreted cautiously and reported with full model details in the Supplementary Materials.

## 3. Results

### 3.1. Fasting Total Ghrelin Concentrations in Women and Men

**Figure 1:**
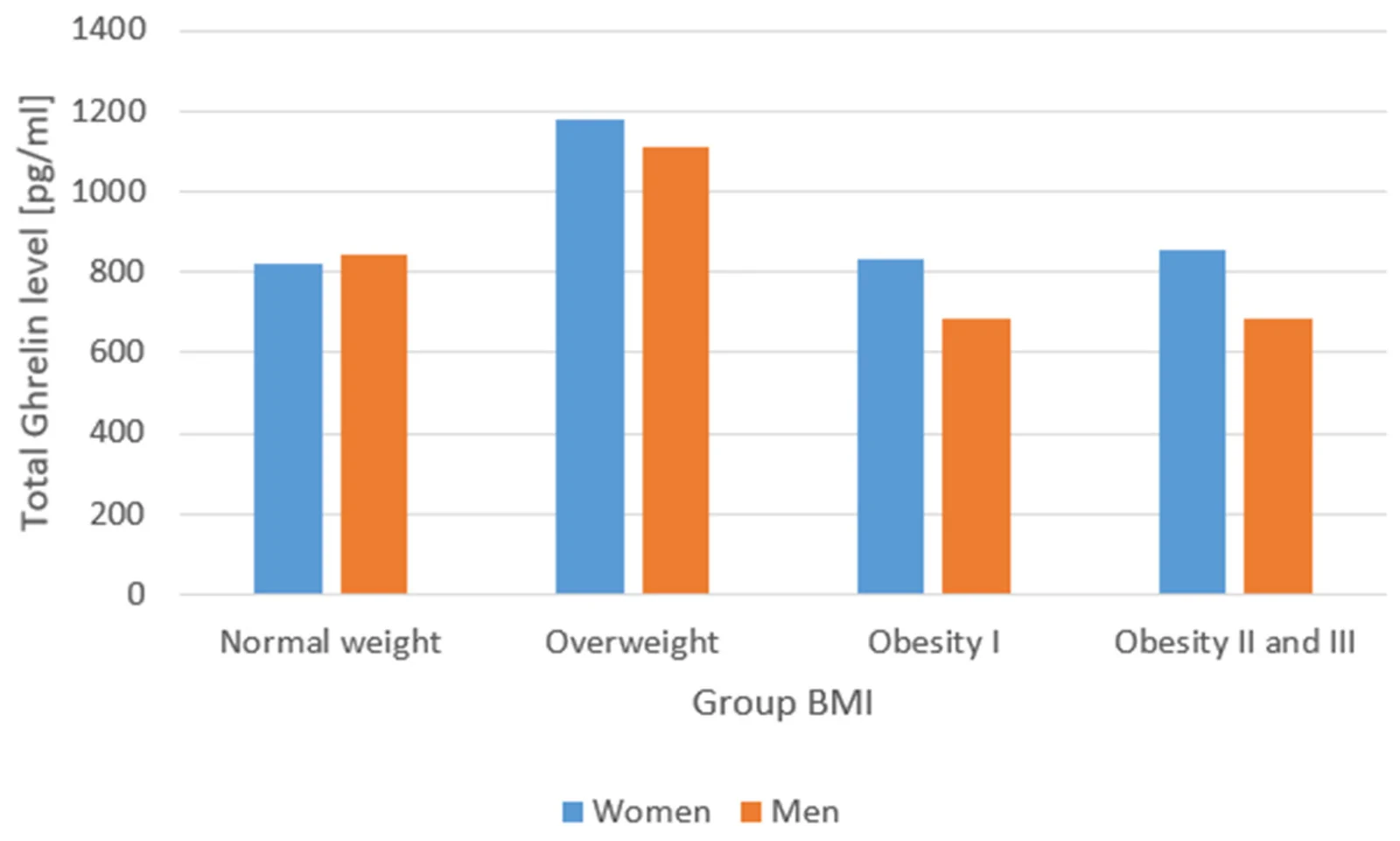
Fasting total ghrelin levels in women and men across BMI categories. Data are presented as mean values. Abbreviations: BMI—body mass index.

Fasting total ghrelin concentrations were comparable between women and men, with no statistically significant difference observed (*p* = 0.190). The median fasting ghrelin concentration was 845.89 pg/mL (Q1–Q3: 594.95–1140.61) in women and 667.81 pg/mL (Q1–Q3: 480.08–1025.11) in men. Across the BMI categories, fasting ghrelin did not differ significantly in women (*p* = 0.530) or in men (*p* = 0.791). In both sexes, the highest fasting ghrelin concentrations were observed in the overweight group (women: 1177.32 pg/mL; men: 1111.48 pg/mL), whereas lower values were noted in class I obesity (women: 834.47 pg/mL; men: 684.93 pg/mL) and class II/III obesity (women: 857.59 pg/mL; men: 686.47 pg/mL). Within each BMI category, fasting ghrelin concentrations remained higher in women than in men, although these sex-specific differences were not statistically significant (normal weight: *p* = 0.842; overweight: *p* = 0.720; class I obesity: *p* = 0.400; class II/III obesity: *p* = 0.661). Fasting total ghrelin concentrations in women and men according to BMI group are presented in Figure 1.

### 3.2. Association Between Habitual Energy and Nutrient Intake and Fasting Total Ghrelin Concentrations

#### 3.2.1. Energy Intake and Fasting Total Ghrelin Concentration

**Figure 2:**
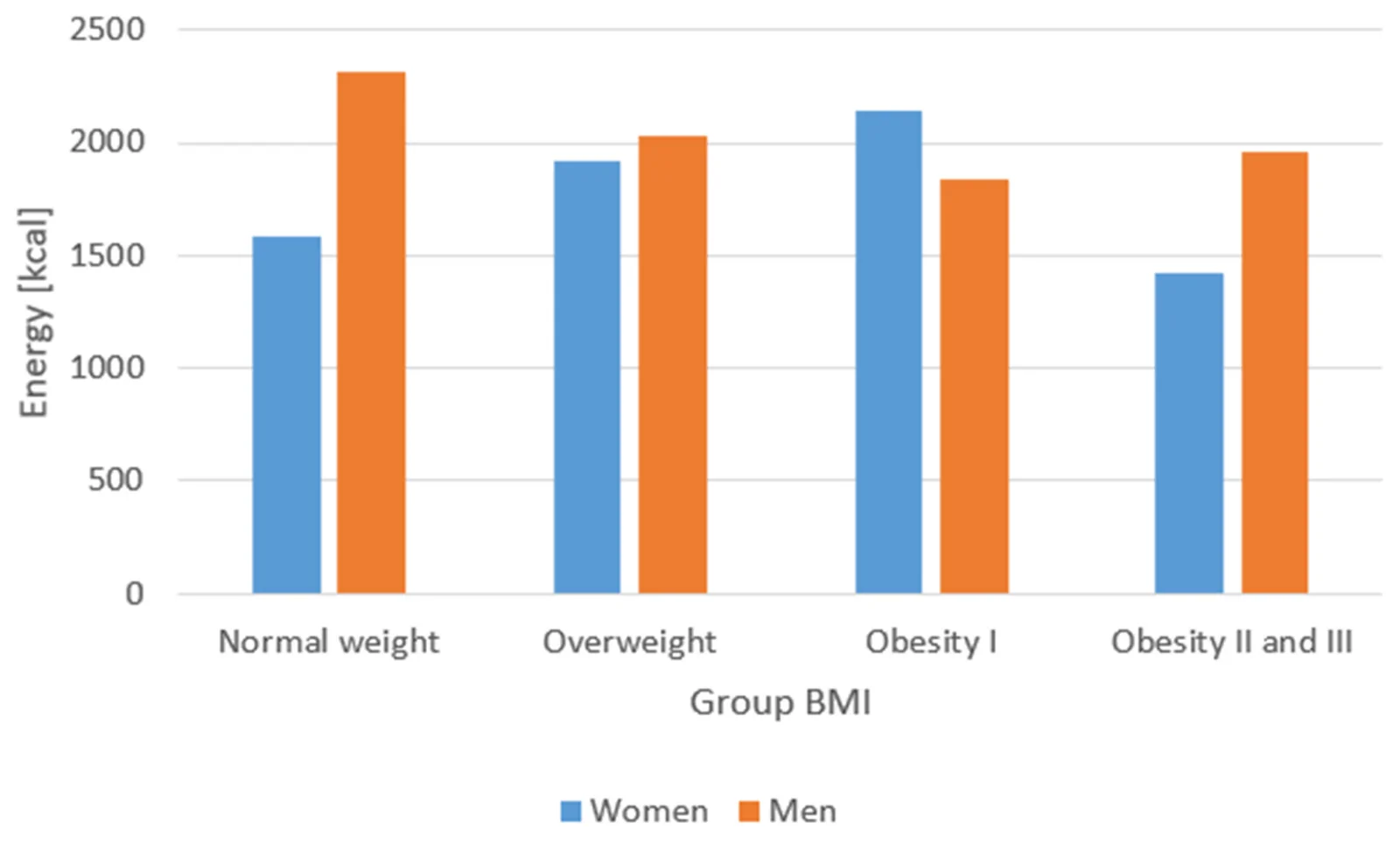
Average daily energy intake in women and men across BMI categories. Data are presented as mean values. Abbreviations: BMI—body mass index.

To assess the association between habitual daily energy intake and fasting total ghrelin concentrations, analyses were performed across four BMI groups, stratified by sex and nutritional status. Mean daily energy intake is presented in Figure 2.

Despite the observed differences in energy intake across BMI groups and between women and men, exploratory Spearman’s rank correlation analyses showed no significant association between daily energy intake and fasting total ghrelin concentrations in either women or men (*p* > 0.05). The detailed distribution of individual energy intake values and fasting total ghrelin concentrations is presented in the Supplementary Figures (Figures S1 and S2). These exploratory correlations were not used as primary inferential analyses, which were based on the multivariable regression models described in Section 3.2.5.

#### 3.2.2. Protein Intake and Fasting Total Ghrelin Concentrations

Mean daily protein intake across BMI and sex groups is presented in Table 3, with individual data provided in the Supplementary Materials (Figures S3–S5). Exploratory Spearman’s rank correlation analyses showed no significant association between daily protein intake and fasting total ghrelin concentrations in either women or men (*p* > 0.05). These exploratory correlations were not used as primary inferential analyses.

#### 3.2.3. Fat Intake and Fasting Total Ghrelin Concentrations

Mean daily fat intake across BMI and sex groups is presented in Table 3, with detailed individual data provided in the Supplementary Materials (Figures S6–S8). Exploratory Spearman’s rank correlation analyses showed no significant association between daily fat intake and fasting total ghrelin concentrations in either women or men (*p* > 0.05). These exploratory correlations were not used as primary inferential analyses.

#### 3.2.4. Carbohydrate Intake and Fasting Total Ghrelin Concentrations

Mean daily carbohydrate intake across BMI and sex groups is presented in Table 3, while individual data are provided in the Supplementary Materials (Figures S9–S11). Exploratory Spearman’s rank correlation analyses showed no significant association between daily carbohydrate intake and fasting total ghrelin concentrations in either women or men (*p* > 0.05). These exploratory correlations were not used as primary inferential analyses, which were based on the multivariable regression models described in Section 3.2.5.

#### 3.2.5. Interaction Between Carbohydrate Intake and BMI Status in Relation to Fasting Total Ghrelin Concentrations

In the multivariable linear regression analyses, habitual carbohydrate intake and BMI status jointly explained fasting total ghrelin concentrations. In the additive model including mean-centered carbohydrate intake and BMI status, the overall model was statistically significant (F(4,71) = 2.76, *p* = 0.034; adjusted R^2^ = 0.086), whereas the association between carbohydrate intake and fasting ghrelin was negative but did not reach conventional significance (β = −1.14, SE = 0.61, *p* = 0.066).

Addition of the carbohydrate intake × BMI-status interaction significantly improved the model fit (F(3,68) = 3.20, *p* = 0.029), increasing the adjusted R^2^ to 0.163. The interaction term for the overweight group was significant (β = −3.71, SE = 1.59, *p* = 0.022), indicating that the carbohydrate–ghrelin slope in overweight participants differed from that in the normal-weight reference group.

Simple-slope analyses showed a significant inverse association between habitual carbohydrate intake and fasting ghrelin in the overweight group (β = −3.17, 95% CI −4.95 to −1.38, *p* = 0.0007), whereas slopes were not significantly different from zero in the normal-weight or obesity groups. However, none of the six pairwise comparisons of slopes remained statistically significant after Holm adjustment for multiple testing. Taken together, these findings suggest that BMI status may modify the association between habitual carbohydrate intake and fasting ghrelin, with the clearest inverse relationship observed in overweight individuals, although specific between-group differences could not be confirmed after correction for multiple comparisons.

### 3.3. Postprandial Changes in Total Ghrelin Concentrations in Women

Total ghrelin concentrations in women were measured in the fasting state and at 60, 120, 180, and 240 min after consumption of the standardized test meal across the four BMI categories. Median fasting ghrelin concentrations tended to be higher in women with overweight and obesity than in women with normal weight, but differences between BMI groups were not statistically significant. Following the test meal, ghrelin concentrations decreased at 60 min in all BMI groups and remained below fasting levels at 120 min, with a gradual return toward the baseline at 180 and 240 min. Detailed median values and interquartile ranges at each time point are presented in Table 4. Exploratory analyses of absolute changes from the baseline (Δ60–0, Δ120–0, Δ180–0, Δ240–0) are presented in the Supplementary Materials (Table S1 and Figure S13).

#### Mixed-Effects Model of Postprandial Ghrelin Trajectories in Women

To formally assess postprandial changes in total ghrelin concentrations in women and account for within-subject correlation of repeated measurements, a linear mixed-effects model with a random intercept for each participant was fitted to log-transformed ghrelin values. The additive model including time and BMI status (log ghrelin ~ time_min + bmi_status + (1 | patient_id)) showed a strong main effect of time, with ghrelin concentrations significantly lower at 60, 120, and 180 min compared with the fasting values and a return toward the baseline at 240 min. BMI status did not significantly predict the overall ghrelin levels after adjustment for time.

In this model, ghrelin concentrations at 60 min were reduced by approximately 40% relative to fasting levels, with smaller but still significant reductions at 120 and 180 min. Women with overweight and class I obesity showed the largest relative decreases within the first 120 min, whereas women with normal weight and higher-grade obesity exhibited more moderate reductions.

An extended model including the time × BMI-status interaction did not significantly improve model fit compared with the additive model (likelihood-ratio test *p* = 0.138), and was therefore considered exploratory. Consequently, the additive mixed-effects model was retained as the primary analytic framework for postprandial ghrelin responses in women.

### 3.4. Postprandial Changes in Total Ghrelin Concentrations in Men

Total ghrelin concentrations in men were measured in the fasting state and at 60, 120, 180, and 240 min after consumption of the standardized test meal across the four BMI categories. Median fasting ghrelin concentrations tended to be higher in men with overweight and obesity than in men with normal weight, although differences between BMI groups were small. Following the test meal, ghrelin concentrations decreased at 60 min in all BMI groups and gradually increased thereafter, approaching fasting values at 240 min. Detailed median values and interquartile ranges at each time point are presented in Table 5. Exploratory analyses of absolute changes from the baseline (Δ60–0, Δ120–0, Δ180–0, Δ240–0) are presented in the Supplementary Materials (Table S2 and Figure S13).

#### Mixed-Effects Model of Postprandial Ghrelin Trajectories in Men

To formally assess postprandial changes in the total ghrelin concentrations in men and account for within-subject correlation of repeated measurements, a linear mixed-effects model with a random intercept for each participant was fitted to log-transformed ghrelin values. The additive model including time and BMI status (log ghrelin ~ time_min + bmi_status + (1 | patient_id)) showed a strong main effect of time, with ghrelin concentrations significantly lower at 60 min compared with the fasting values, followed by a gradual increase at 120, 180, and 240 min. BMI status did not significantly predict the overall ghrelin levels after adjustment for time.

In this model, ghrelin concentrations at 60 min were reduced by approximately 30–35% relative to the fasting levels, with a progressive return toward the baseline thereafter. The temporal pattern of postprandial ghrelin response was similar across BMI groups, with no evidence of distinct group-specific trajectories.

An extended model including the time × BMI-status interaction did not significantly improve the model fit compared with the additive model (likelihood-ratio test *p* = 0.219), and was therefore considered exploratory. Consequently, the additive mixed-effects model was retained as the primary analytic framework for postprandial ghrelin responses in men.

### 3.5. Comparison of Postprandial Changes in Total Ghrelin Concentrations in Women and Men

**Figure 3:**
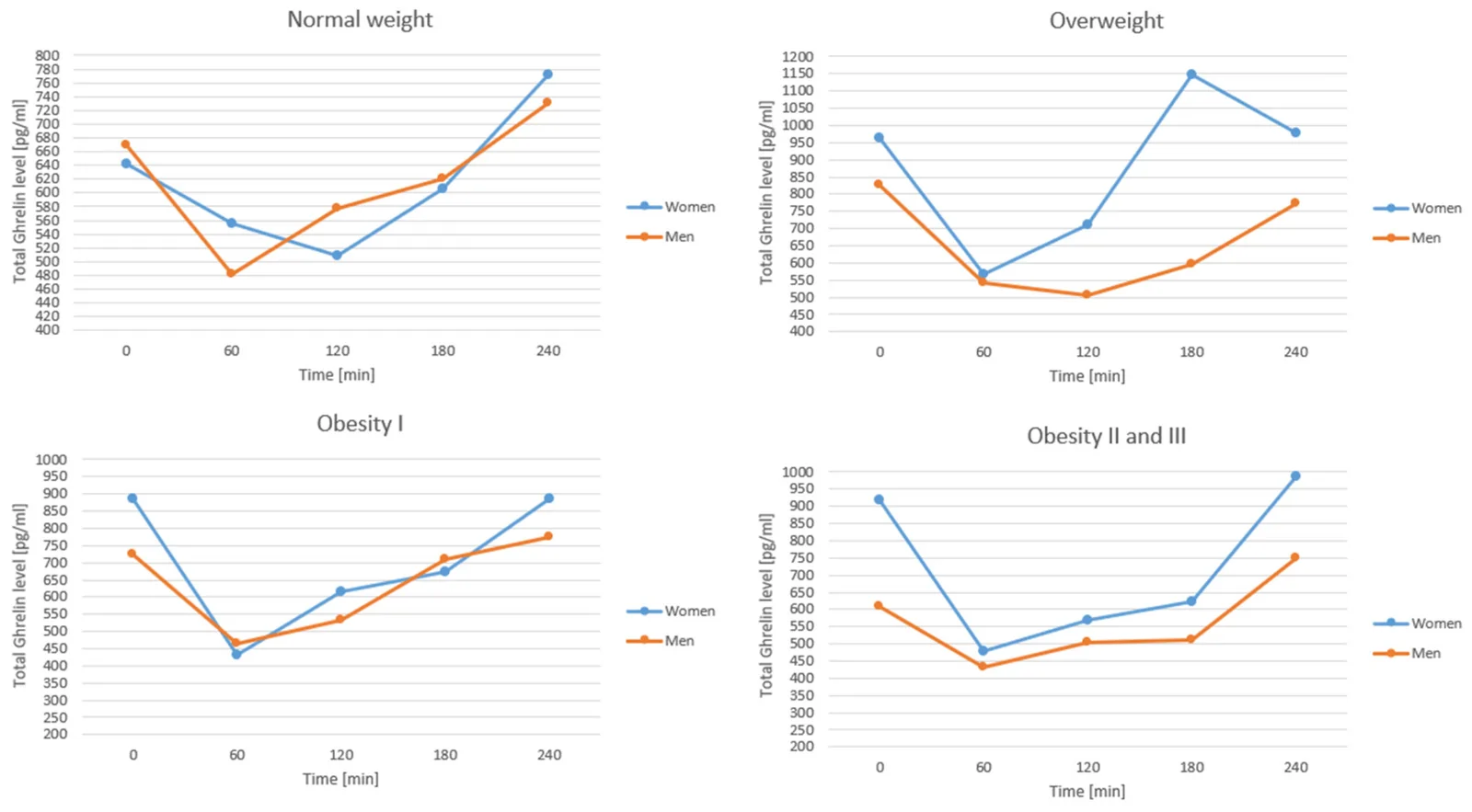
Postprandial changes in total ghrelin concentrations in women and men across the four BMI categories following consumption of the standardized test meal. Abbreviations: BMI—body mass index. Data are presented as time-dependent trajectories (0–240 min).

Postprandial trajectories of the total ghrelin concentrations in women and men were compared descriptively across the four BMI categories. Median ghrelin concentrations in both sexes decreased at 60 min after meal consumption and gradually increased thereafter, approaching fasting values at 240 min. Although the timing and magnitude of these changes varied slightly between women and men within some BMI groups, the overall temporal pattern was broadly similar. Time-dependent trajectories for women and men in each BMI category are presented in Figure 3.

In all BMI groups, both women and men exhibited a decline in median total ghrelin concentrations at 60 min after meal consumption, followed by a gradual recovery toward the baseline values. The temporal pattern of these changes differed slightly according to sex and BMI category. Among the participants with normal body weight, the nadir in women occurred at 120 min, whereas in men, it was observed at 60 min. In the overweight group, women showed an increase in ghrelin concentration by 120 min after the initial decline at 60 min, while in men, lower concentrations persisted up to 120 min. In contrast, in the class I obesity and class II/III obesity groups, postprandial ghrelin profiles were similar in women and men, with the greatest decline observed at 60 min and a gradual increase thereafter. Overall, although some differences were observed in the normal-weight and overweight groups, the postprandial total ghrelin response was broadly comparable between women and men.

### 3.6. Factors Associated with Fasting Total Ghrelin Concentrations: Multiple Regression Analysis

To identify factors associated with fasting total ghrelin concentrations, parsimonious multivariable linear regression models were fitted separately in women and men. Fasting total ghrelin concentration was entered as the dependent variable, while selected body composition and fat distribution parameters, resting metabolic rate, and mean energy and macronutrient intake were considered as potential predictors. Model selection was based on statistical significance and interpretability, with the final models presented in Table 6 and Table 7.

In women, the parsimonious multivariable regression model identified BMI and gynoid fat percentage as significant predictors of fasting total ghrelin concentrations. Higher fasting ghrelin levels were associated with higher BMI and a lower proportion of fat mass in the gynoid region. The android-to-gynoid ratio and resting metabolic rate were retained in the final model but were not statistically significant. The final regression model is presented in Table 6.

In men, the parsimonious multivariable regression model identified gynoid fat percentage as a significant predictor of fasting total ghrelin concentrations. Higher fasting ghrelin levels were associated with a lower proportion of fat mass in the gynoid region. Mean daily carbohydrate intake was retained in the final model and showed a tendency toward an inverse association with fasting ghrelin concentrations, although this relationship did not reach statistical significance. The final regression model is presented in Table 7.

## 4. Discussion

In the present study, we evaluated the associations of sex, nutritional status, and current energy and macronutrient intake, calculated using the Dieta 6.0 software, with fasting total ghrelin concentrations and with postprandial changes in ghrelin following consumption of a standardized liquid meal. No significant associations were found between fasting total ghrelin concentrations and recently assessed energy or macronutrient intake based on 3-day food records, suggesting that short-term dietary variability may be less relevant for fasting ghrelin than longer-term characteristics of body composition and fat distribution. At the same time, the regression analyses indicated that selected body composition and fat distribution parameters may be associated with fasting ghrelin concentrations. These findings should be interpreted with caution, particularly in women, given the modest effect sizes and limited sample size. Overall, the results suggest that body composition parameters may be more closely associated with interindividual variability in fasting ghrelin concentrations than recently assessed energy and macronutrient intake.

The lack of significant differences in fasting total ghrelin concentrations between women and men is consistent with some of the available data [33,34,35], although the literature points to a potential influence of sex hormones on appetite regulation and metabolic responses [33,34]. Estradiol may modulate central nervous system sensitivity to metabolic signals, including ghrelin signaling [34] and some studies have reported higher ghrelin concentrations in women as well as variation across the menstrual cycle [36]. It has also been emphasized that a substantial proportion of experimental research on ghrelin physiology has been conducted in male animal models, which limits a comprehensive assessment of sex-related differences [12]. In the present study, the relatively narrow age range of the participants, the similar representation of BMI categories in both sexes, and the measurement of total ghrelin without differentiation between acylated and desacyl ghrelin may have limited the ability to detect sex-related differences.

In the present analysis, no significant associations were found between the mean daily intake of energy, protein, fat, or carbohydrates and fasting total ghrelin concentrations. This finding is consistent with the observations of Ellis et al., who showed that an 8-week adaptation to diets differing in carbohydrate and fat content did not significantly affect fasting ghrelin or GLP-1 concentrations, nor their responses to a standardized meal [37]. Similarly, Votruba et al. reported that morning ghrelin concentrations remained stable despite short-term overfeeding and did not predict subsequent spontaneous energy intake [38]. Paul et al. also found no effect of energy intake or dietary macronutrient composition on preprandial ghrelin concentrations [39]. These findings contrast with the well-documented influence of the composition of an individual meal on the acute postprandial ghrelin response. As reported by Koliaki et al., the type of macronutrient consumed may affect both the magnitude and duration of postprandial ghrelin suppression, while the characteristics of this response may also depend on nutritional status and the study protocol used [40]. Taken together, these observations suggest that fasting total ghrelin concentrations may be less strongly related to recent energy and macronutrient intake, whereas short-term postprandial changes in ghrelin may be more sensitive to meal composition.

The present findings can be discussed in the context of theoretical models of body weight regulation, including the set-point and settling-point frameworks, which describe how hormonal and metabolic signals may contribute to short- and long-term energy balance. The classical set-point model proposes the existence of feedback mechanisms that counteract deviations in body weight and energy stores from a relatively stable level, partly through compensatory hormonal changes occurring in response to energy deficit [24,25,26]. Ghrelin may participate in these adaptive mechanisms, as its concentrations change in response to both short-term energy deprivation and longer-term alterations in body weight. Studies of short-term fasting also indicate significant changes in the GH/IGF-1 axis, particularly increased GH secretion, which is not necessarily accompanied by a parallel increase in IGF-1 [41,42,43].

In contrast, the settling-point model proposes that body weight stabilizes as a result of dynamic interactions between metabolic mechanisms, the environment, and eating behavior [29,30,44]. More recent studies suggest that evidence for a rigid, single set point in humans remains limited and that body weight regulation is likely to be more dynamic [44,45]. In this context, ghrelin may be viewed as one of the signals involved in both the short-term regulation of food intake and long-term adaptation to changes in energy balance and body weight [12,27,28]. The lack of significant associations between recently assessed energy and macronutrient intake and fasting total ghrelin concentrations in our study is consistent with the hypothesis that fasting ghrelin may be more closely related to longer-term characteristics of energy balance and body composition than to short-term variability in dietary intake.

Following consumption of the standardized test meal, women in all BMI groups showed a decrease in total ghrelin concentrations during the first 60–120 min, consistent with a typical physiological postprandial response. The greatest changes from the baseline were observed in women with overweight and class I obesity, whereas smaller decreases were seen in women with normal body weight and those with class II/III obesity. Thus, the observed pattern did not indicate a simple linear relationship between the degree of obesity and the magnitude of ghrelin suppression. Previous studies have shown that postprandial ghrelin regulation may be altered in obesity. Le Roux et al. reported attenuated meal energy-dependent ghrelin suppression in individuals with obesity [46], whereas a meta-analysis by Aukan et al. demonstrated lower total ghrelin concentrations both in the fasting state and postprandially in individuals with obesity compared with those without obesity [35]. At later time points, 180 and 240 min after the meal, differences between BMI groups in our study were no longer statistically significant, indicating that the greatest between-group variability occurred during the early postprandial phase.

In men, the postprandial response was more homogeneous, with no significant differences between BMI groups at any of the analyzed time points. These findings suggest that in the men included in our study, the postprandial ghrelin response varied less according to BMI category. Differences between women and men may be related to sex-specific regulation of appetite and metabolism, including the modulatory effects of sex hormones on ghrelin signaling [31,47,48]; however, this mechanism cannot be directly established from the present study.

In the regression analysis performed in women, higher fasting total ghrelin concentrations were associated with higher BMI and a lower proportion of fat mass in the gynoid region. These associations should, however, be interpreted with caution, as the overall regression model did not reach statistical significance. In men, a lower proportion of gynoid fat was the only factor significantly associated with fasting total ghrelin concentrations. These findings suggest that not only the total amount of adipose tissue, but also its regional distribution, may be associated with interindividual variability in fasting ghrelin concentrations, which is consistent with previous observations linking adipose tissue distribution patterns with hormonal responses [32,35,49]. Taken together, our findings suggest that fasting total ghrelin concentrations may be more closely related to body composition and fat distribution parameters than to recently assessed energy and macronutrient intake [12,37,38,39].

The present study has several limitations. First, the study population was restricted to individuals aged 40–60 years, which limits the generalizability of the findings to younger populations. Second, total ghrelin was measured without distinguishing between its acylated and desacylated forms. Dietary intake was assessed using 3-day food records, which may not fully reflect habitual long-term dietary patterns and are subject to reporting bias. In addition, only one type of standardized liquid meal was used, limiting the ability to assess whether the observed postprandial response would differ according to meal composition or physical form. Finally, menstrual cycle phase and menopausal status were not taken into account in women, which may have influenced the ghrelin concentrations and postprandial responses. Moreover, due to the small sample sizes within sex- and BMI-stratified subgroups, the present analysis should be considered preliminary and exploratory, and the findings should be interpreted with caution given the limited statistical power.

Despite these limitations, the findings provide further insight into the factors associated with fasting and postprandial ghrelin concentrations. The results suggest that fasting total ghrelin may be more closely associated with body composition and regional fat distribution than with recently assessed energy and macronutrient intake. They also indicate that sex and adipose tissue distribution should be considered when investigating individual variability in ghrelin responses and when designing future nutritional interventions targeting appetite regulation.

In conclusion, the present findings suggest that fasting total ghrelin concentrations may be more closely related to body composition and regional fat distribution than to short-term variability in energy and macronutrient intake. Although some sex-related differences in the timing of postprandial changes were observed descriptively, these patterns should not be interpreted as statistically confirmed and require verification in studies specifically designed to assess sex-by-time interactions. Overall, the interpretation of ghrelin concentrations should consider body composition, regional fat distribution, and sex, while recognizing that short-term dietary intake may play a limited role in explaining fasting ghrelin variability. These findings provide a basis for further research into the role of ghrelin in appetite and energy balance regulation and its potential utility as a biomarker of metabolic and energy-balance disturbances.

## 5. Conclusions

1. Fasting total ghrelin concentrations were not significantly associated with recently assessed energy or macronutrient intake, whereas selected body composition and fat distribution parameters showed associations with fasting ghrelin concentrations.
2. Fasting total ghrelin concentrations did not differ significantly between women and men. BMI-related differences in the early postprandial response were observed in women but not in men.
3. In women, the greatest postprandial decreases in total ghrelin concentrations were observed in the overweight and class I obesity groups, indicating BMI-related variability in the early postprandial ghrelin response.
4. Regional fat distribution, particularly gynoid fat, may contribute to interindividual variability in fasting ghrelin concentrations; however, the findings in women should be interpreted cautiously because the overall regression model did not reach statistical significance.
5. Further studies incorporating acylated and desacylated ghrelin measurements, different meal compositions, larger study populations, and the assessment of sex-related hormonal factors are needed to clarify the determinants of fasting and postprandial ghrelin responses.

## Figures and Tables

**Table 1:** Anthropometric characteristics and body composition parameters of the female and male study participants.

Parameter	Women (*n* = 38)		Men (*n* = 38)		*p* Values
	Median	Q1–Q3	Median	Q1–Q3	
Age (years)	45.50	42.00–53.00	48.00	46.00–54.00	0.106
Height (cm)	165.00	163.00–169.00	180.00	176.00–185.00	<0.001
Weight (kg)	78.00	69.00–87.00	99.00	84.00–104.80	<0.001
BMI (kg/m^2^)	29.60	24.90–34.00	30.50	25.30–35.00	0.405
Android fat (%)	49.10	30.80–54.70	43.30	35.80–47.10	0.086
Gynoid fat (%)	44.10	38.70–47.60	30.30	24.70–35.20	<0.001
VAT area (cm^2^)	127.00	49.00–186.00	194.50	100.00–291.00	<0.001
SAT area (cm^2^)	237.00	115.00–293.00	179.00	113.00–249.00	<0.001
A/G ratio	1.02	0.81–1.14	1.32	1.18–1.42	<0.001

Values are expressed as the median and interquartile range (Q1–Q3); differences between women and men were assessed using the Mann–Whitney U test. Statistical significance was set at *p* < 0.05. Abbreviations: BMI—body mass index, %bf—percent body fat, VAT—visceral adipose tissue, SAT—subcutaneous adipose tissue, A/G—android-to-gynoid fat ratio.

**Table 2:** Composition of the standardized test meal.

Nutritional Value	Nutridrink Protein (Portion 187.5 mL)
Energy	459.4 kcal
Carbohydrates	47.1 g
% of energy	41%
Sugars	25.7 g
Lactose	<0.66 g
Fat	18 g
% of energy	35%
Saturated fat	1.6 g
Protein	27.4 g
% of energy	24%
Dietary fiber	0 g

**Table 3:** Mean energy and nutrient intake across BMI groups, stratified by sex.

Parameter	Normal Weight		Overweight		Obesity I		Obesity II and III	
	Women	Men	Women	Men	Women	Men	Women	Men
Energy (kcal)	1583.71	2314.65	1920.51	2035.10	2142.36	1842.45	1417.72	1961.15
Protein (g)	84.35	106.54	87.76	102.80	97.34	97.20	70.75	105.83
Fat (g)	56.00	92.89	66.25	102.02	77.26	59.94	50.74	75.11
Carbohydrates (g)	187.70	272.03	241.71	230.72	266.58	220.78	174.27	222.15

**Table 4:** Total ghrelin concentrations in the fasting state and at 60, 120, 180, and 240 min after consumption of the standardized test meal in women across the four BMI groups.

Parameter	Women Normal Weight (*n* = 10)		Women Overweight (*n* = 10)		Women Obesity I (*n* = 9)		Women Obesity II and III (*n* = 9)		*p* Value
	Median	Q1–Q3	Median	Q1–Q3	Median	Q1–Q3	Median	Q1–Q3	
Total Ghrelin (0 min)	642.71	525.92–866.16	964.35	750.33–1419.74	885.85	840.80–1013.75	920.14	368.44–1140.61	0.530
Total Ghrelin (60 min)	555.27	382.27–616.72	568.18	389.18–876.33	431.05	414.78–577.44	479.85	262.69–574.46	0.619
Total Ghrelin (120 min)	508.31	422.16–633.20	710.63	394.80–1005.11	615.89	504.09–663.23	567.59	311.74–809.44	0.930
Total Ghrelin (180 min)	605.25	488.07–869.17	1147.33	429.37–1311.89	672.09	603.29–743.07	623.63	377.86–1031.64	0.865
Total Ghrelin (240 min)	771.83	519.07–917.56	976.63	569.08–1699.60	885.16	737.27–1020.13	988.40	418.64–1100.15	0.856

Values are expressed as the median and interquartile range (Q1–Q3); Differences between BMI groups were assessed using the Kruskal–Wallis test. Statistical significance was set at *p* < 0.05.

**Table 5:** Total ghrelin concentrations in the fasting state and at 60, 120, 180, and 240 min after consumption of the standardized test meal in men across the four BMI groups.

Parameter	Men Normal Weight (*n* = 9)		Men Overweight (*n* = 9)		Men Obesity I (*n* = 10)		Men Obesity II and III (*n* = 10)		*p* Value
	Median	Q1–Q3	Median	Q1–Q3	Median	Q1–Q3	Median	Q1–Q3	
Total Ghrelin (0 min)	670.06	355.82–791.44	826.41	480.08–1686.70	722.81	479.50–912.43	608.03	534.95–818.02	0.791
Total Ghrelin (60 min)	481.95	269.64–591.20	540.66	331.99–971.65	464.21	357.92–621.13	431.55	378.97–460.09	0.910
Total Ghrelin (120 min)	577.00	443.33–1026.87	505.18	403.52–1068.33	533.39	464.91–678.60	505.01	387.45–538.37	0.882
Total Ghrelin (180 min)	621.37	505.63–853.73	595.12	527.13–1031.64	709.32	455.81–772.17	512.24	431.47–713.19	0.697
Total Ghrelin (240 min)	730.61	539.91–1059.05	772.77	527.48–1318.85	773.92	710.83–944.22	749.57	432.35–879.07	0.930

Values are expressed as the median and interquartile range (Q1–Q3); Differences between BMI groups were assessed using the Kruskal–Wallis test. Statistical significance was set at *p* < 0.05.

**Table 6:** Multiple regression model of factors associated with fasting total ghrelin concentrations in women.

Variable	β	SE	*p*
Gynoid fat (%)	–0.8736	0.3487	0.0172
BMI (kg/m^2^)	1.0442	0.4771	0.0356
Android/Gynoid ratio	–0.3836	0.2372	0.1150
Resting metabolic rate (kcal/day)	–0.3373	0.2467	0.1806

Abbreviations: β, standardized regression coefficient; SE, standard error; BMI, body mass index.

**Table 7:** Multiple regression model of factors associated with the fasting total ghrelin concentrations in men.

Variable	β	SE	*p*
Gynoid fat (%)	–0.3477	0.1585	0.0351
Mean carbohydrate intake (g/day)	–0.2695	0.1585	0.0981

Abbreviations: β—standardized regression coefficient; SE—standard error; *p*—significance level.

## Data Availability

The data presented in this study are available on request from the corresponding author. The data are not publicly available due to privacy and ethical reasons.

## Supplementary Materials

The following supporting information can be downloaded at: https://www.mdpi.com/article/10.3390/nu18193271/s1, Figure S1: Relationship between fasting total ghrelin concentration and daily energy intake in women across four BMI categories. Each point represents an individual participant. These analyses are exploratory and do not form part of the confirmatory statistical framework. Figure S2: Relationship between fasting total ghrelin concentration and daily energy intake in men across four BMI categories. Each point represents an individual participant. These analyses are exploratory and do not form part of the confirmatory statistical framework. Figure S3: Relationship between fasting total ghrelin concentration and daily protein intake in women across four BMI categories. Each point represents an individual participant. These analyses are exploratory and do not form part of the confirmatory statistical framework. Figure S4: Relationship between fasting total ghrelin concentration and daily protein intake in men across four BMI categories. Each point represents an individual participant. These analyses are exploratory and do not form part of the confirmatory statistical framework. Figure S5: Average daily protein intake in women and men across BMI categories. Data are presented as mean values. These analyses are exploratory and do not form part of the confirmatory statistical framework. Figure S6: Relationship between fasting total ghrelin concentration and daily lipids intake in women across four BMI categories. Each point represents an individual participant. These analyses are exploratory and do not form part of the confirmatory statistical framework. Figure S7: Relationship between fasting total ghrelin concentration and daily lipids intake in men across four BMI categories. Each point represents an individual participant. These analyses are exploratory and do not form part of the confirmatory statistical framework. Figure S8: Average daily lipids intake in women and men across BMI categories. Data are presented as mean values. These analyses are exploratory and do not form part of the confirmatory statistical framework. Figure S9: Relationship between fasting total ghrelin concentration and daily carbohydrates intake in women across four BMI categories. Each point represents an individual participant. These analyses are exploratory and do not form part of the confirmatory statistical framework. Figure S10: Relationship between fasting total ghrelin concentration and daily carbohydrates intake in men across four BMI categories. Each point represents an individual participant. These analyses are exploratory and do not form part of the confirmatory statistical framework. Figure S11: Average daily carbohydrates intake in women and men across BMI categories. Data are presented as mean values. These analyses are exploratory and do not form part of the confirmatory statistical framework. Figure S12: Changes in total ghrelin concentrations relative to fasting values in women across four BMI groups following consumption of the standardized test meal. Data are presented as median changes from baseline (Δ60–0, Δ120–0, Δ180–0, and Δ240–0). These analyses are exploratory and were not part of the primary mixed-effects model. Figure S13: Changes in total ghrelin concentrations relative to fasting values in men across four BMI groups following consumption of the standardized test meal. Data are presented as median changes from baseline (Δ60–0, Δ120–0, Δ180–0, and Δ240–0). Differences between BMI groups were assessed using the Kruskal–Wallis test. These analyses are exploratory and were not part of the primary mixed-effects model. Table S1: Changes in total ghrelin concentrations relative to fasting values (Δ60–0, Δ120–0, Δ180–0, and Δ240–0) in women across four BMI groups. These analyses are exploratory and were not part of the primary mixed-effects model. Table S2: Changes in total ghrelin concentrations relative to fasting values (Δ60–0, Δ120–0, Δ180–0, and Δ240–0) in men across four BMI groups. These analyses are exploratory and were not part of the primary mixed-effects model.
