# Supplementary material for: Do Nutritional Status and Dietary Intake Influence the Ghrelin Response?
Source: Nutrients. 2026 Oct 4;18(19):3271. doi: 10.3390/nu18193271 (PMC13667174; doi:10.3390/nu18193271)
Supplement: Supplementary file 1 [file nutrients-18-03271-s001.zip › nutrients-4578845-supplementary.pdf]

Supplementary Material – Exploratory Analyses

The analyses presented in Figures S1–S11 are exploratory and were not included in the primary statistical models. They illustrate individual-level variability in dietary intake and fasting ghrelin concentrations and should be interpreted cautiously. The main inferential results are based on multivariable regression models described in Section 3.2.5 of the main manuscript.

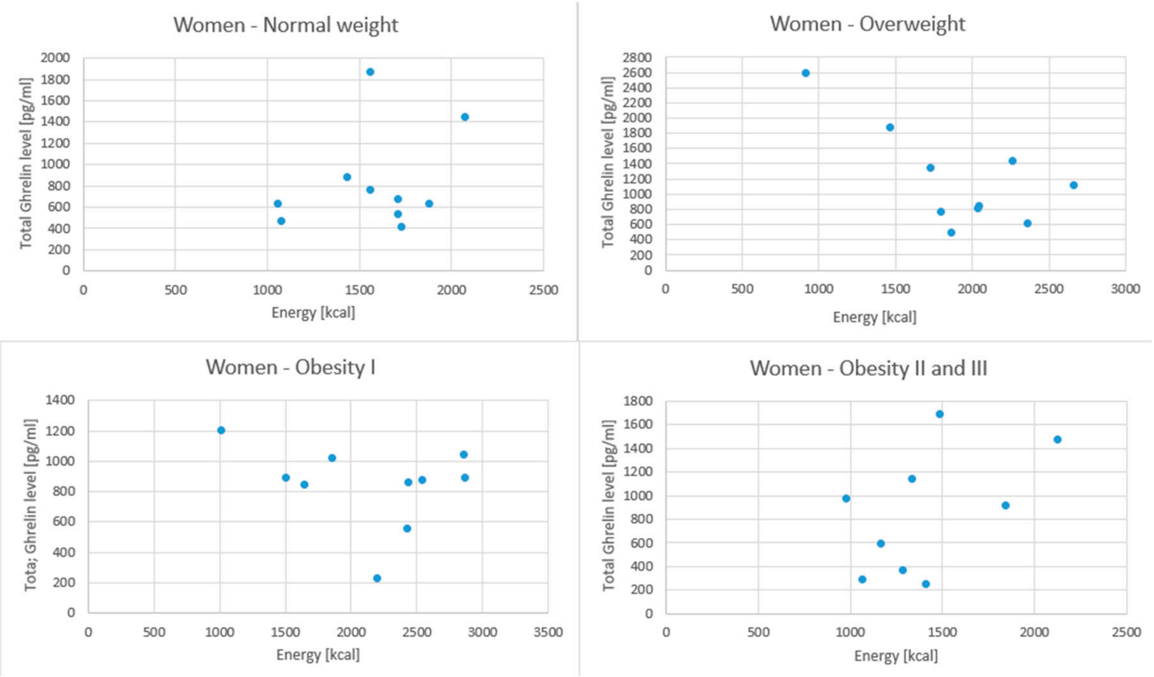

Abbreviations: BMI — body mass index. Associations were evaluated using Spearman's rank correlation.

Figure S1. Relationship between fasting total ghrelin concentration and daily energy intake in women across four BMI categories. Each point represents an individual participant. These analyses are exploratory and do not form part of the confirmatory statistical framework.

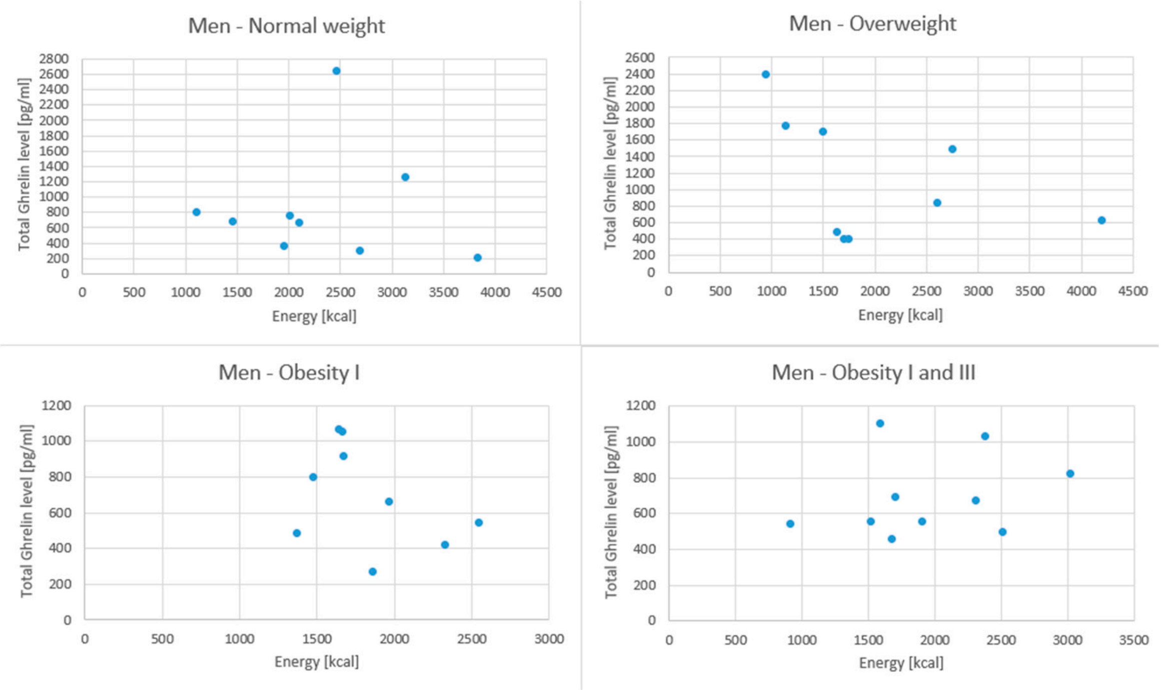

Abbreviations: BMI — body mass index. Associations were evaluated using Spearman's rank correlation.

Figure S2. Relationship between fasting total ghrelin concentration and daily energy intake in men across four BMI categories. Each point represents an individual participant. These analyses are exploratory and do not form part of the confirmatory statistical framework.

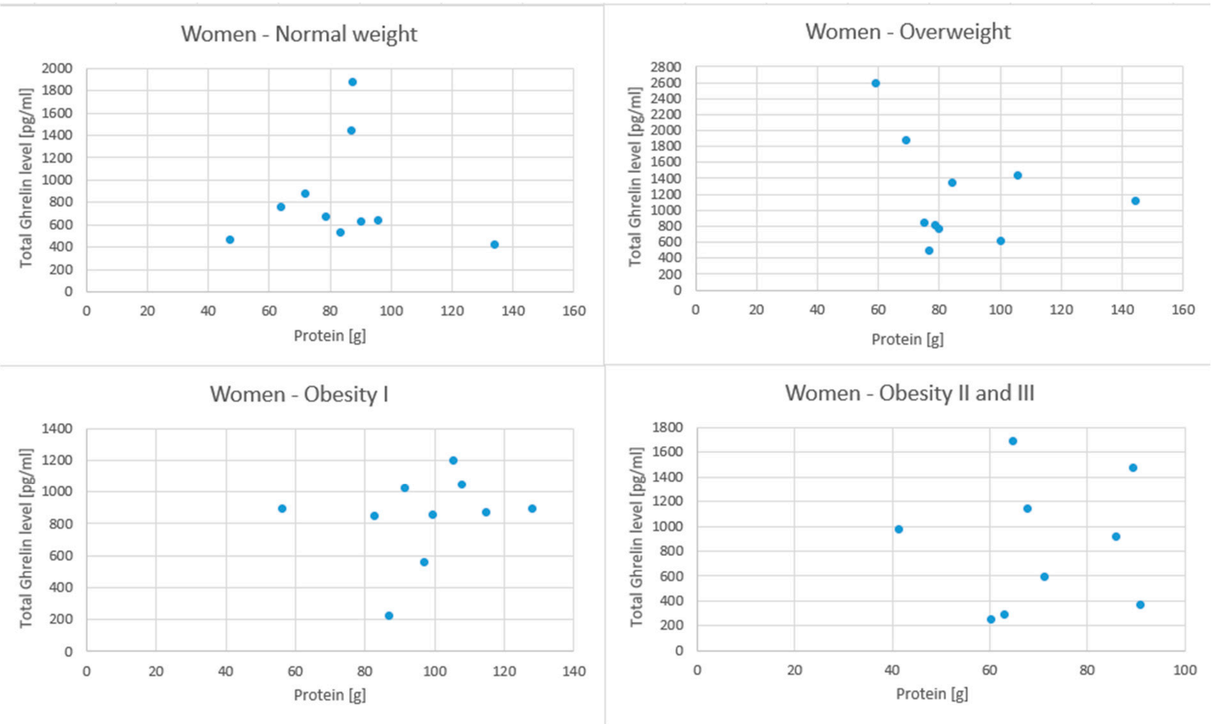

Abbreviations: BMI — body mass index. Associations were evaluated using Spearman's rank correlation.

Figure S3. Relationship between fasting total ghrelin concentration and daily protein intake in women across four BMI categories. Each point represents an individual participant. These analyses are exploratory and do not form part of the confirmatory statistical framework.

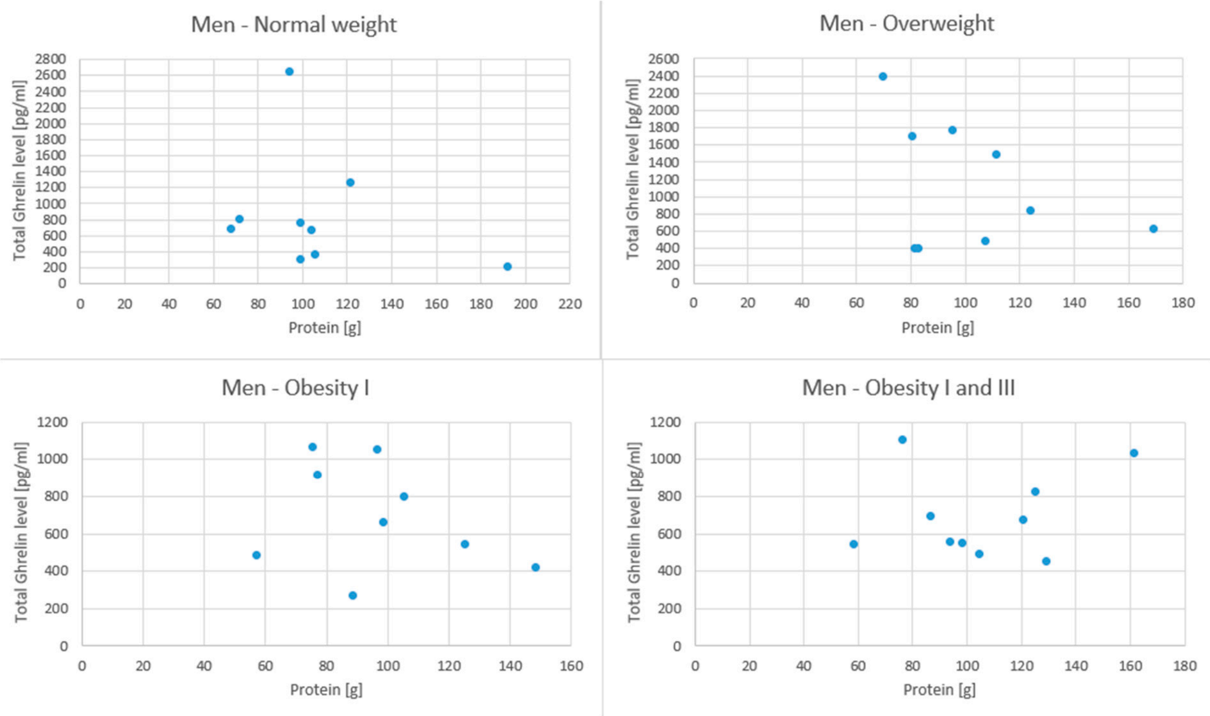

Abbreviations: BMI — body mass index. Associations were evaluated using Spearman's rank correlation

Figure S4. Relationship between fasting total ghrelin concentration and daily protein intake in men across four BMI categories. Each point represents an individual participant. These analyses are exploratory and do not form part of the confirmatory statistical framework.

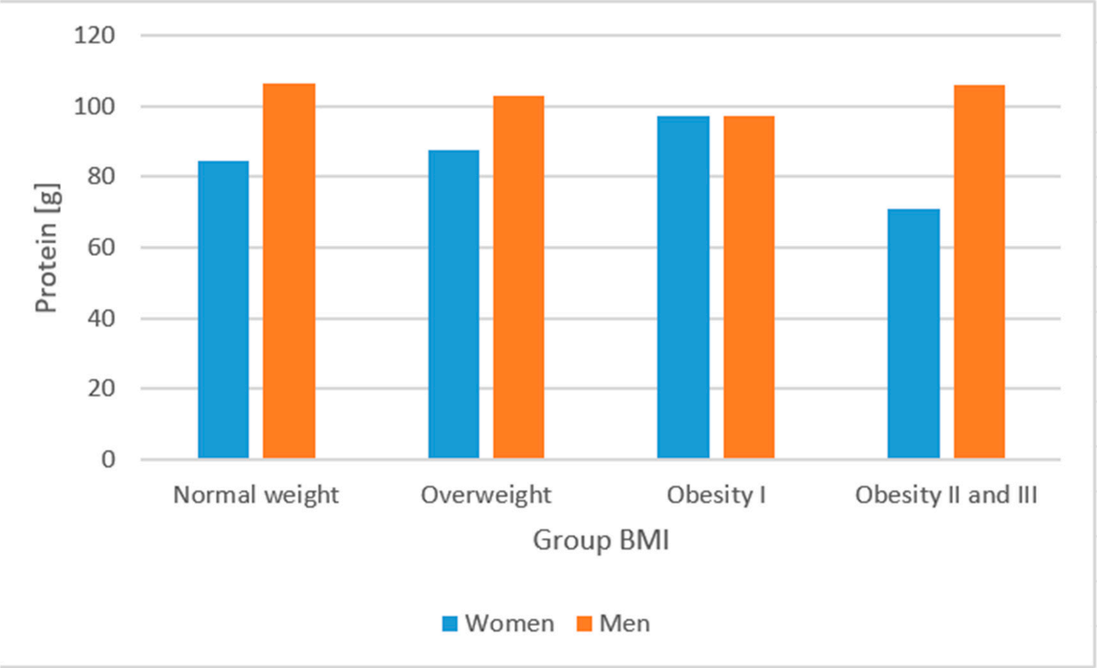

Abbreviations: BMI — body mass index.

Figure S5. Average daily protein intake in women and men across BMI categories. Data are presented as mean values. These analyses are exploratory and do not form part of the confirmatory statistical framework.

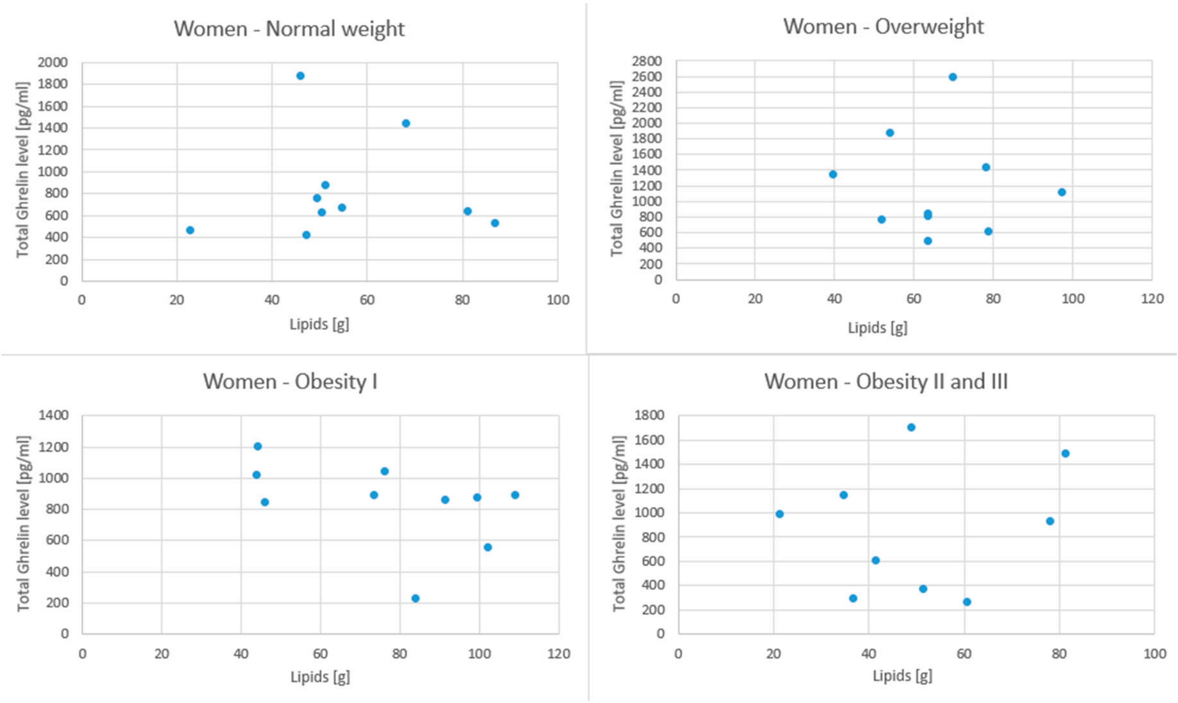

Abbreviations: BMI — body mass index. Associations were evaluated using Spearman's rank correlation.

Figure S6. Relationship between fasting total ghrelin concentration and daily lipids intake in women across four BMI categories. Each point represents an individual participant. These analyses are exploratory and do not form part of the confirmatory statistical framework.

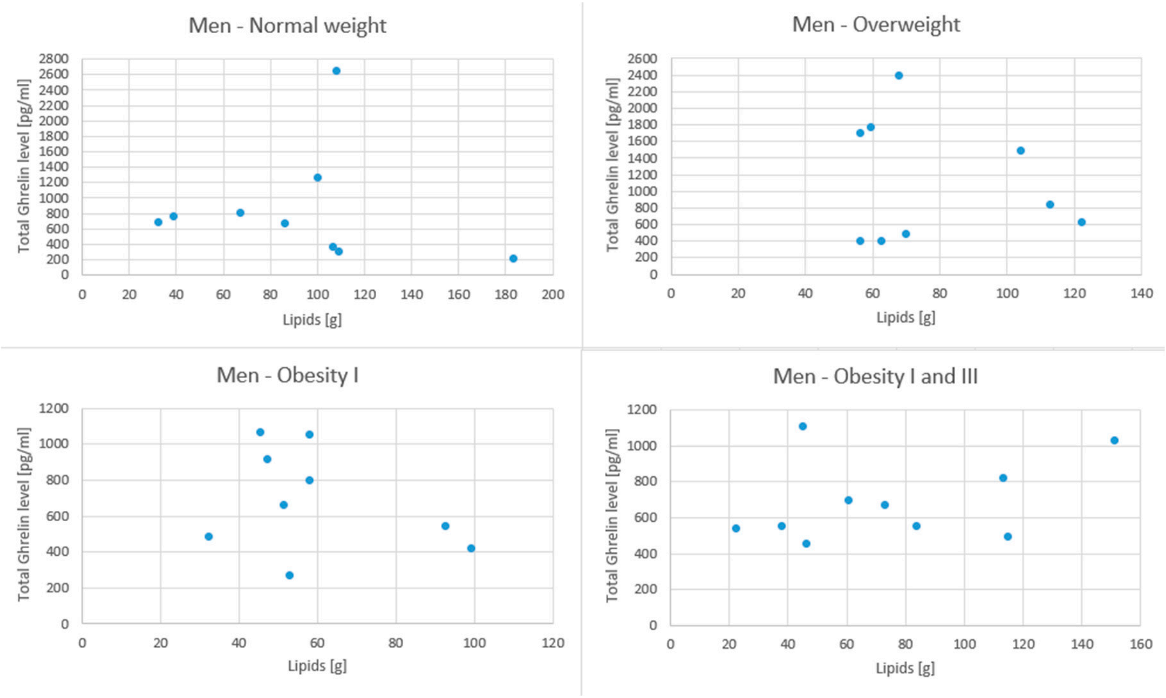

Abbreviations: BMI — body mass index. Associations were evaluated using Spearman's rank correlation.

Figure S7. Relationship between fasting total ghrelin concentration and daily lipids intake in men across four BMI categories. Each point represents an individual participant. These analyses are exploratory and do not form part of the confirmatory statistical framework.

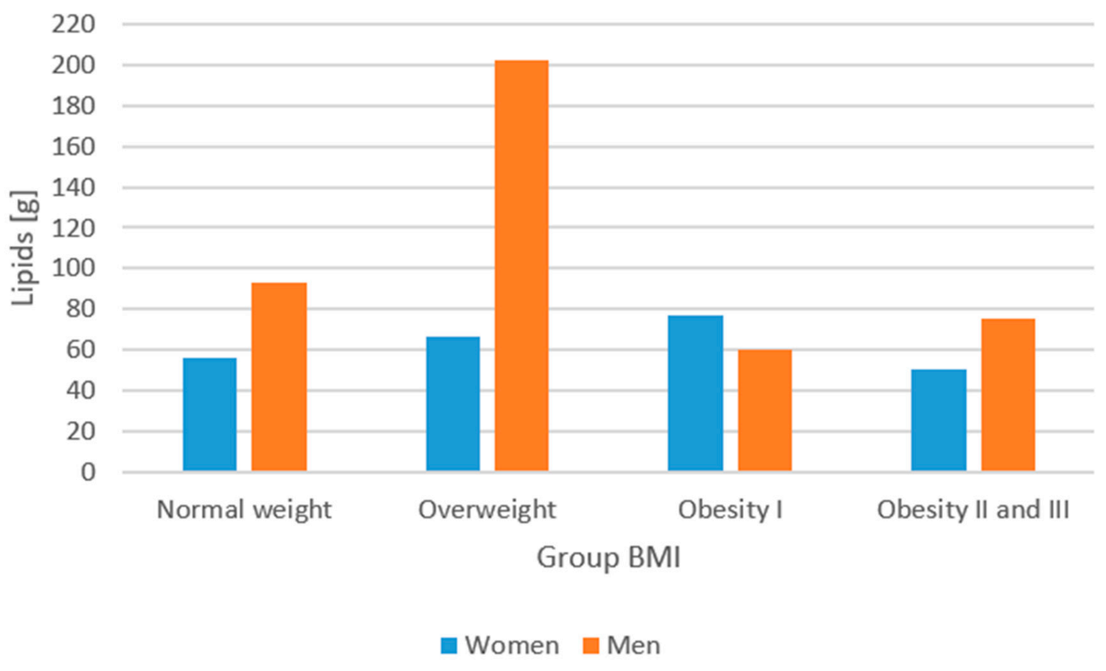

Abbreviations: BMI — body mass index.

Figure S8. Average daily lipids intake in women and men across BMI categories. Data are presented as mean values. These analyses are exploratory and do not form part of the confirmatory statistical framework.

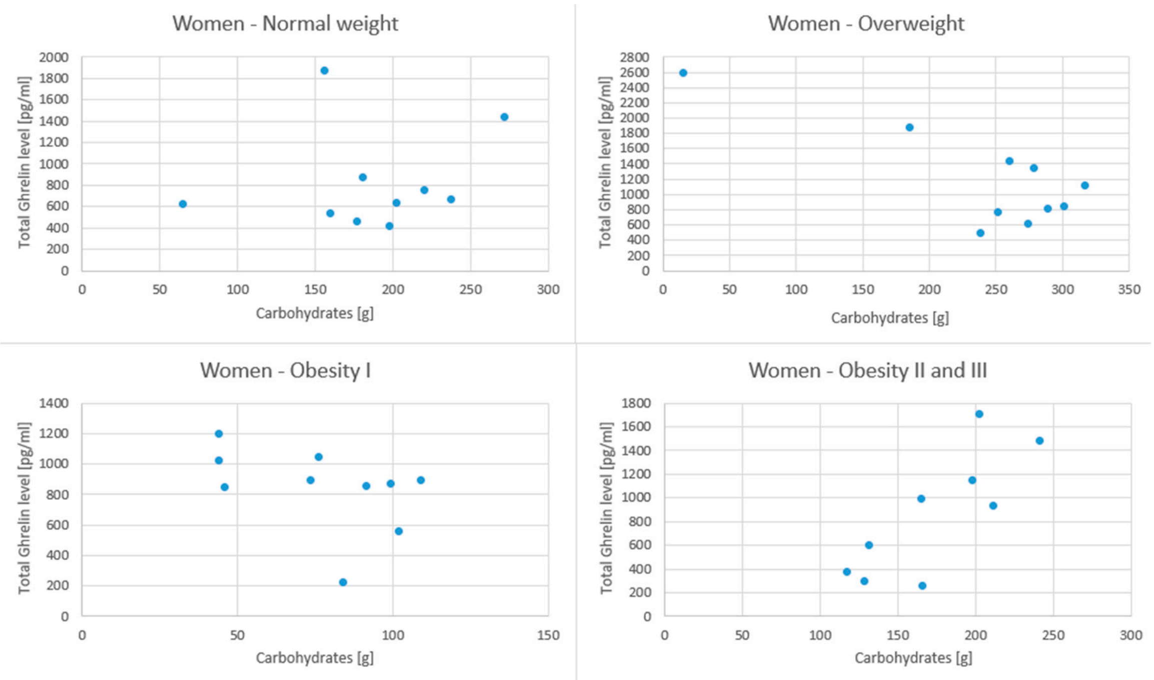

Abbreviations: BMI — body mass index. Associations were evaluated using Spearman's rank correlation.

Figure S9. Relationship between fasting total ghrelin concentration and daily carbohydrates intake in women across four BMI categories. Each point represents an individual participant. These analyses are exploratory and do not form part of the confirmatory statistical framework.

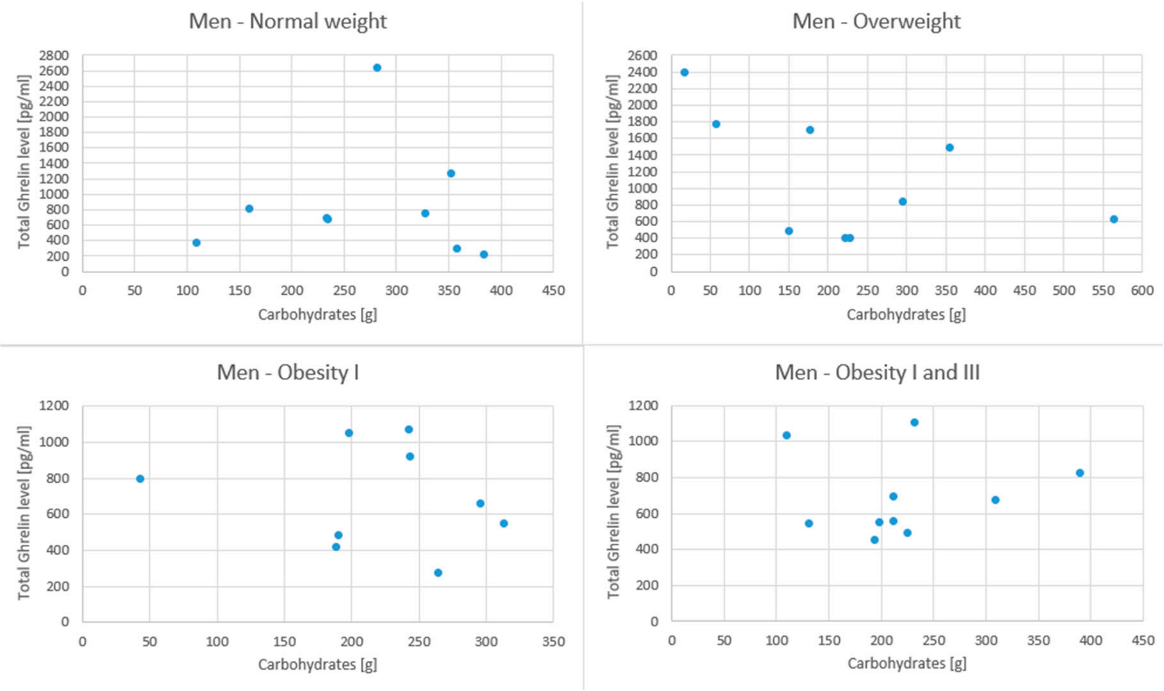

Abbreviations: BMI — body mass index. Associations were evaluated using Spearman's rank correlation.

Figure S10. Relationship between fasting total ghrelin concentration and daily carbohydrates intake in men across four BMI categories. Each point represents an individual participant. These analyses are exploratory and do not form part of the confirmatory statistical framework.

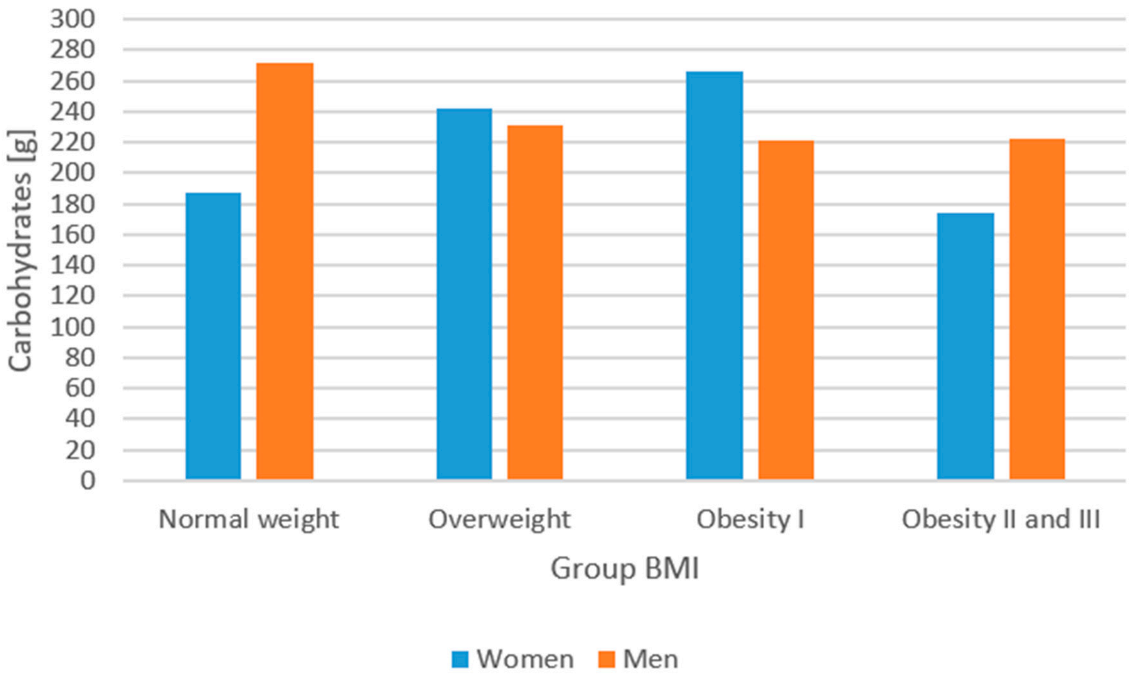

Abbreviations: BMI — body mass index.

Figure S11. Average daily carbohydrates intake in women and men across BMI categories. Data are presented as mean values. These analyses are exploratory and do not form part of the confirmatory statistical framework.

Table S1. Changes in total ghrelin concentrations relative to fasting values (Δ60–0, Δ120–0, Δ180–0, and Δ240–0) in women across four BMI groups. These analyses are exploratory and were not part of the primary mixed-effects model.

Parameter	Women Normal weight (n=10)		Women Overweight (n=10)		Women Obesity I (n=9)		Women Obesity II and III (n=9)		p Value
	Median	Q1-Q3	Median	Q1-Q3	Median	Q1-Q3	Median	Q1-Q3	
ΔTotal Ghrelin (60-0 min)	-121.53	-256.47- -27.28	-367.75	-767.59- -315.83	-400.72	-419.92- -348.57	-152.25	-405.69- -105.75	0.039
ΔTotal Ghrelin (120-0 min)	-128.62	-235.49- -33.77	-389.30	-444.50- -266.54	-325.20	-336.71- -196.10	-79.27	-226.02- -42.75	0.040
ΔTotal Ghrelin (180-0 min)	-55.07	-230.27-179.16	-252.32	-338.42- -26.30	-183.46	-187.51- -100.72	25.40	-52.78-126.34	0.175
ΔTotal Ghrelin (240-0 min)	-1.07	-131.55-192.03	-170.39	-225.82-2.63	-1.54	-14.49-117.28	44.56	-28.02-167.12	0.344

Values are presented as median and interquartile range (Q1–Q3). Differences between BMI groups were assessed using the Kruskal–Wallis test. Statistical significance was set at p < 0.05. Abbreviations: BMI, body mass index; Δ, change relative to baseline (0 min).

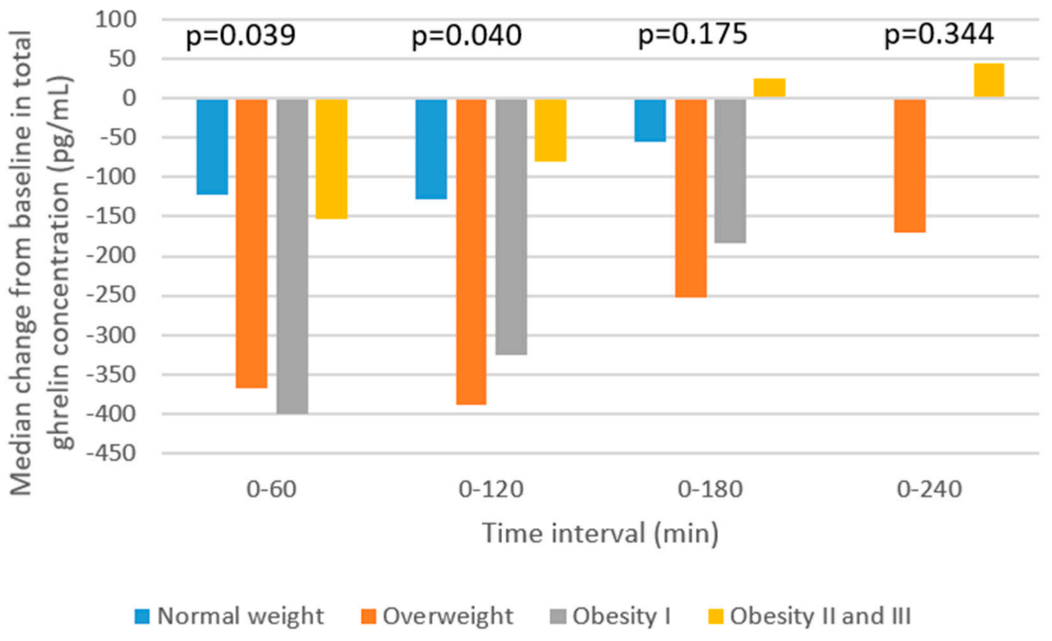

Differences between BMI groups were assessed using the Kruskal–Wallis test. Statistical significance was set at p < 0.05. Abbreviations: BMI — body mass index;

Figure S12. Changes in total ghrelin concentrations relative to fasting values in women across four BMI groups following consumption of the standardized test meal. Data are presented as median changes from baseline (Δ60–0, Δ120–0, Δ180–0, and Δ240–0). These analyses are exploratory and were not part of the primary mixed-effects model.

Table S2. Changes in total ghrelin concentrations relative to fasting values (Δ60–0, Δ120–0, Δ180–0, and Δ240–0) in men across four BMI groups. These analyses are exploratory and were not part of the primary mixed-effects model.

**Parameter**	**Men Normal weight (n=9)**		**Men Overweight (n=9)**		**Men Obesity I (n=10)**		**Men Obesity II and III (n=10)**		**p Value**
	Median	Q1-Q3	Median	Q1-Q3	Median	Q1-Q3	Median	Q1-Q3	
ΔTotal Ghrelin (60-0 min)	-177.22	-305.71- -87.78	-252.09	-281.40- -131.90	-235.40	-335.45- -112.88	-251.37	-306.73- -117.81	0.906
ΔTotal Ghrelin (120-0 min)	-87.70	-174.76- 15.61	-122.56	-205.24- -43.40	-131.00	-205.86- -28.06	-185.70	-278.11- -42.31	0.621
ΔTotal Ghrelin (180-0 min)	-50.26	-104.83- 21.22	37.06	-157.54- 185.00	21.13	-154.02- 90.84	-87.74	-208.61- 72.67	0.828
ΔTotal Ghrelin (240-0 min)	14.19	-60.84- 169.43	47.39	-48.57- 114.75	84.87	-79.56- 327.87	-6.67	-46.08- 197.48	0.929

Values are expressed as the median and interquartile range (Q1–Q3); Differences between BMI groups were assessed using the Kruskal–Wallis test. Statistical significance was set at p < 0.05. Abbreviations: BMI—body mass index. Δ—change relative to baseline (0 min).

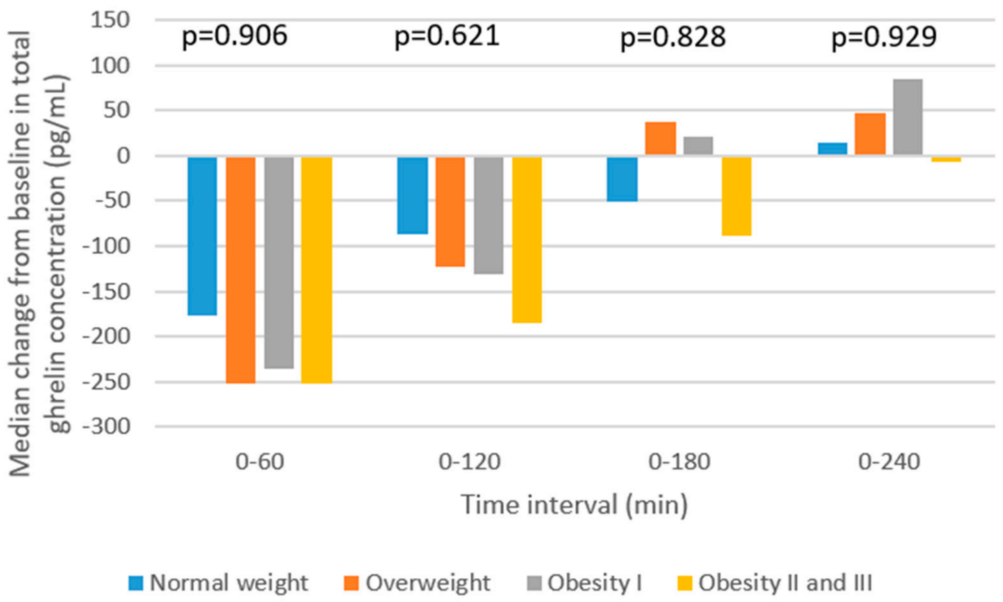

Differences between BMI groups were assessed using the Kruskal–Wallis test. Statistical significance was set at p < 0.05. Abbreviations: BMI — body mass index;

Figure S13. Changes in total ghrelin concentrations relative to fasting values in men across four BMI groups following consumption of the standardized test meal. Data are presented as median changes from baseline (Δ60–0, Δ120–0, Δ180–0, and Δ240–0). Differences between BMI groups were assessed using the Kruskal–Wallis test. These analyses are exploratory and were not part of the primary mixed-effects model.
